# Prof. Austin Flint, Invited to a Public Dinner by the Profession of Buffalo

**Published:** 1868-08

**Authors:** Austin Flint


### Editorial Department.

### Prof. Austin Flint, invited to a Public Dinner by the Profession of Buffalo.

The following correspondence will be perused with much pleasure by many of our readers. It sufficiently explains itself and shows how warmly attached are the members of the medical profession to their old associate, Prof. Flint, who during his twenty years' residence in Buffalo gained the respect and confidence of the public, and in remarkable degree the respect and confidence of the profession, not only here, but throughout the entire country. His present world-wide reputation as a teacher and author, commenced while a resident of Buffalo. This is source of pride to his numerous professional friends in this city, who are ready on every occasion to do him honor.

BUFFALO, August 24th, 1868.

To Prof. Austin Flint, M. D.:

Dear Sir:—Having learned that you are in Buffalo, the guest of Prof. White, we, the undersigned, your friends and professional associates, in token of regard for your high attainment and private worth, and for the purpose of renewing our old and pleasantly remembered personal acquaintance, beg you to accept an invitation to a public entertainment which we desire to give on the occasion of your re-visiting the place of your former residence and the scene of so many of your early professional achievements.

Hoping that the gratification of our wishes may be consistent with your other engagements, we wait your designation of the *time* when you will afford us this pleasure.

Yours, very respectfully, etc., etc.

Sandford Eastman,
J. R. Lothrop,
George P. Eddy,
T. T. Lockwood,
E. Tobie,
Thos. F. Rochester,
John J. Trowbridge,
J. I. Richards,
J. C. Greene,
John Cronyn,
P. H. Strong,
C. B. Hutchins,
David E. Chace,
Edward Little,
J. B. Samo,
M. H. Shaw,
C. F. A. Nichell,
George Ayer,
J. F. Miner,
C. C. Wyckoff,
W. Gould,
S. W. Wetmore,
Charles Winne,
Thos. M. Johnson,
S. F. Mixer,
William Ring,
James S. Smith,
G. E. Mackay,
Conrad Diehl,
G. Whitaker,
J. E. King,
John Hauenstein,
H. Nichell,
C. C. F. Gay,
James P. White.
Edward Storck.

BUFFALO, August 25th, 1868.

My dear friends and former professional associates:

Your most kind communication has been received, and I cannot undertake to give utterance to the feelings which it occasions. Revisiting the city where more than twenty years of my professional life were passed, I derive a pleasure greater than I can express in the renewal of personal intercourse with members of the profession with whom I was so long and so pleasantly associated. In declining to meet you at a public entertainment—which I am compelled to do in consequence of engagements already made for the few days of my visit—I beg to assure you that I appreciate most fully the honor conferred upon me by your invitation, and that I shall ever hold in grateful remembrance the kindness which has prompted it. I trust I shall be able to return personally to each of you my thanks before I leave the city; and, with my warmest wishes in behalf of the medical profession of Buffalo, and for the welfare of all of its members to whom I am greatly indebted for kindness in times past, as well as now, I remain,

Very sincerely yours,

AUSTIN FLINT.

